# Creation of the ECHO Idaho Podcast: Tutorial and Pilot Assessment

**DOI:** 10.2196/55313

**Published:** 2025-03-21

**Authors:** Ryan Wiet, Madeline P Casanova, Jonathan D Moore, Sarah M Deming, Russell T Baker Jr

**Affiliations:** 1 WWAMI Medical Education Program, Idaho Office of Underserved and Rural Medical Research, University of Idaho, 875 Perimeter Drive, Moscow, ID, 83843, United States

**Keywords:** Project ECHO, ECHO Idaho, medical education, medical training, medication teaching, medical knowledge, rural health care, rural medicine, underserved population, underserved people, substance use, substance use disorder, SUD, drug abuse, drug use, alcoholism, addiction, pain, behavioral health, podcast, webinar

## Abstract

**Background:**

Project ECHO (Extension for Community Health Outcomes) is an innovative program that uses videoconferencing technology to connect health care providers with experts. The model has been successful in reaching health care providers in rural and underserved areas and positively impacting clinical practice. ECHO Idaho, a replication partner, has developed programming that has increased knowledge and confidence of health care professionals throughout the state of Idaho, United States. Although the ECHO model has a demonstrated ability to recruit, educate, and train health care providers, barriers to attending Project ECHO continuing education (CE) programs remain. The asynchronous nature of podcasts could be used as an innovative medium to help address barriers to CE access that health care professionals face. The ECHO Idaho “Something for the Pain” podcast was developed to increase CE accessibility to rural and frontier providers, while upscaling their knowledge of and competence to treat and assess substance use disorders, pain, and behavioral health conditions.

**Objective:**

This paper describes the creation and preliminary assessment of the ECHO Idaho “Something for the Pain” podcast.

**Methods:**

Podcast episodes consisted of interviews with individuals as well as didactic lectures. Audio from these recordings were edited for content and length and then professionally reviewed by subject matter experts (eg, featured episode speakers). Target audiences consisted of health care providers and community members interested in behavioral health and substance use disorders. Metrics on podcast listeners were assessed using SoundCloud’s RSS feed, continuing education survey completion, and iECHO.

**Results:**

The ECHO Idaho “Something for the Pain” podcast’s inaugural season comprised 14 episodes with 626 minutes of CE material. The podcast series received a total of 2441 listens from individuals in 14 different cities across Idaho, and 63 health care providers listened and claimed CE credits. The largest professional group was social workers (n=22; 35%).

**Conclusions:**

We provide preliminary evidence that podcasts can be used to provide health care providers with opportunities to access CE material. Health care providers listened to and claimed CE credits from the ECHO Idaho “Something for the Pain” podcast. Project ECHO programs should consider creating podcasts as an additional platform for disseminating ECHO material.

## Introduction

Project ECHO (Extension for Community Health Outcomes), founded by the University of New Mexico, provides accessible education and specialty training to rural and underserved professionals through an all-teach, all-learn model that uses interactive video conferencing (ie, Zoom technology) [1]. In 2018, through a collaboration between the University of Idaho WWAMI (Washington, Wyoming, Alaska, Montana, Idaho) Regional Medical Education Program and the Idaho Department of Health and Welfare Statewide Healthcare Innovation Plan, ECHO Idaho became the only Project ECHO replication partner in the state of Idaho [2]. Since 2018, ECHO Idaho has provided rural and underserved communities with educational opportunities and cultivated a community of health care professional learners across the state. Researchers assessing the Project ECHO model have found that attendees increase their knowledge, clinical skills, and confidence, thus increasing the quality of care for patients in rural and underserved communities [1-10]. Health care professionals participating in the ECHO Idaho series have similarly reported increases in both their knowledge and in their confidence to provide specialty care for their patients [2,11].

Despite the myriad documented benefits of the Project ECHO model, challenges relevant to maximizing attendance and increasing overall reach of the program (eg, recruiting participants from rural areas) still exist [2-4,12]. For example, scheduling conflicts and time constraints have been reported as barriers to attending ECHO sessions [5,10,13]. Although some hubs have modified the ECHO model (eg, changed session lengths), no conclusive changes have been recommended and minimal research has been conducted to assess how modifications to the model could positively impact and enhance provider training [10,13]. Other models of continuing education (CE) (eg, e-learning) have been reported to be effective in changing knowledge, self-efficacy, and skills [14]. However, the effect of such models on professional and clinical practice, as well as patient outcomes, remains largely unknown.

Thus, exploring and researching novel or innovative platforms to disseminate information and increase clinical skills for health care providers, while subsequently decreasing potential barriers to attendance, are necessary. One proposed approach is the use of a podcast platform to deliver Project ECHO materials. A podcast may be an effective, interactive, and cost-efficient way to deliver Project ECHO programming while addressing some barriers reported by participants. For example, the asynchronous nature of podcasts would allow providers the flexibility to engage with the material on their own time, and to pause and resume listening when needed. Research has reported that podcasts can be an impactful model to train and enhance professional development [15-17]. Additionally, due to the COVID-19 pandemic dramatically changing the landscape of health care and education [18], the use of podcasts to deliver medical education curricula has increased [19-22]. Researchers have found podcasts increase participant knowledge and self-efficacy while also indirectly impacting professional and clinical practice [19-22]; however, the full scope of the impact of educational podcasts remains unknown [16].

To test the efficacy of the podcast model for delivering medical education, ECHO Idaho developed the “Something for the Pain” podcast, with support from the Idaho WWAMI Medical Education Program and in partnership with the Valley County Opioid Response Project [23]. The podcast was meant to increase CE accessibility for rural and frontier providers, while upscaling their knowledge of and competence to treat and assess behavioral health conditions. The purpose of this paper was to present the development and implementation process of the ECHO Idaho “Something for the Pain” podcast’s inaugural season, as well as to discuss the preliminary findings regarding the reach of the podcast, lessons learned, and proposed future uses of this innovative platform.

## Methods

### Program Development and Implementation

#### Program Description

Due to the increased need for more specialty training related to behavioral health, opioids, pain, and substance use disorder (SUD), in 2021, the ECHO Idaho “Something for the Pain” podcast was developed as an innovative approach to disseminate ECHO Idaho materials. The inaugural season contained 14 episodes that presented best-practices and resources for behavioral health, opioid use disorder, and SUD prevention, treatment, and recovery specific to Idaho. The podcast was free to access, and eligible providers were able to obtain no-cost CE credits for listening to an episode.

#### Episode Development

The ECHO model learning framework typically includes two sections: (1) didactic lectures and (2) a case-based presentation and discussion. To mirror the framework of the ECHO model, the 14 podcast episodes were primarily broken down into two parts: (1) interviews with individuals and (2) didactic presentations. The interviews were with health care professionals and community members who had first-hand experience and knowledge of evidence-based practices and resources pertinent to the Idaho context. The didactic presentations were taken from previously recorded ECHO Idaho sessions across 4 series (ie, Behavioral Health in Primary Care; Opioids, Pain and Substance Use Disorders; Counseling Techniques for Substance Use Disorders; and Viral Hepatitis and Liver Care). Audio from these recordings were edited for content and length and then professionally reviewed by subject matter experts. The subject matter experts also assisted with generating CE-eligible episode assessment questions that could be used to gauge audience engagement.

#### Audience Recruitment

The primary intended audience for the podcast was health care providers and community members with an interest in SUD and behavioral health. Initial recruitment occurred by using ECHO Idaho’s network of prior ECHO Idaho session attendees, which, at the time of the first episode’s release in May 2021, consisted of approximately 3000 diverse Idaho health care professionals (eg, those who held an MD/DO, PA, NP, MCSW, or LCPC). Members of the ECHO Idaho staff invited participants from the pre-existing ECHO network to listen to the podcast as an additional means of earning CE credit at their convenience. Additionally, ECHO staff developed a targeted marketing campaign that involved personal and bulk emails, newsletter announcements, weekly announcements, paid advertisements, social media posts, print bulk postcard mailings, as well as advertising on other podcast programs. The podcast was housed on SoundCloud, which increased its accessibility through user search engines and algorithms.

### Podcast Data Metrics

Listener engagement was tracked using SoundCloud’s RSS feed. The feed provided click metrics (ie, how many listens occurred) for each podcast episode consumed within a specified timeframe. Eeds, an electronic CE management system, was used to track CE credits claimed following episode and assessment completion. Additionally, to gain insight into listener experience, participants were required to answer a user experience question in Eeds. Lastly, individuals interested in listening to the podcast could register on the ECHO Idaho website. Registration information collected included demographic questions like profession, credentials, primary practice location, sex, and age. iECHO, a web-based proprietary program and management software database, was used to manage participant demographic data (ECHO Institute, University of New Mexico) entered on the registration form.

### Data Analysis

Data were exported from Eeds and iECHO and a descriptive statistical analysis was performed using SPSS, version 28 (IBM Corp).

### Ethical Considerations

The project was certified as exempt (protocol #23‐150) by the Institutional Review Board at the University of Idaho. Data were deidentified for the purposes of analysis, and informed consent was obtained from all participants prior to their involvement in the study.

## Results

### RSS Feed

The first season of the ECHO Idaho “Something for the Pain” podcast included 14 episodes (released May 2021 to June 2022); 13 episodes were related to perinatal SUD, and 1 bonus episode gave a brief history of Project ECHO and Vandal Theory (Table 1). The average length of an episode was 45 (SD 11.9) minutes and the average number of listens per episode was 188 (SD 46.5) (Table 1). The season provided a total of 626 minutes of CE material available for perpetual access. The podcast was released on the streaming platforms SoundCloud, Apple Podcasts, Google Podcasts, Spotify, Sticher, and iHeartRadio. As of April 19, 2023, the initial season of the podcast had garnered over 2000 listeners from various parts of the United States, with most of the listeners based in Idaho.

**Table 1.** Information on each episode including release date, title, length, and total listens of each episode.

Release date	Episode	Length (minutes)	Listens
5/11/2021	Episode 1: Framework for Addiction as Disease (feat. Craig Lodis, PhD)	31	286
5/17/2021	Episode 2: State of Substance Use in Idaho (feat. Amy Jeppesen, LCSW, ACADC)	47	212
6/3/2021	Bonus Episode: Project ECHO Origin Story & The Vandal Theory (feat. Sanjeev Arora, MD)	37	N/A^a^
7/1/2021	Episode 3: De-escalation Techniques and the Valley County Court Services’ Diversion Program (feat. Abby Abbondondalo and Skip Clapp)	45	159
7/11/2021	Episode 4: Harm Reduction and Valley County’s Opioid Response Project (feat. Brenda Hoyt, NP, Courtney Boyce and Shelly Hitt)	47	165
7/23/2021	Episode 5: Motivational Interviewing and Donnelly’s The Change Clinic (feat. Deb Thomas and Barbara Norton)	60	191
8/9/2021	Episode 6: LaDessa Foster Talks Levels of Care in Substance Use Disorder Treatment (feat. LaDessa Foster)	32	164
8/23/2021	Episode 7: Monica Forbes Talks SMART Recovery, Stigma and Re-entering Society Post-Incarceration (feat. Monica Forbes)	35	178
9/1/2021	Episode 8: Marjorie, Wilson Talks Idaho’s Syringe Service Programs (feat. Marjorie, Wilson and Ian Trosoyer, DNP)	51	185
9/13/2021	Episode 9: LaDessa Foster Talks Managing Clinical Services for Patients and Providers (feat. LaDessa Foster)	30	274
3/2/2022	Episode 10: Lindsay Brown Talks Peer Recovery Supports (feat. Lindsay Brown)	55	168
3/25/2022	Episode 11: Talking Telehealth in SUD Care | McCall Mobile Medicine (feat. Drew Holliday, MSW)	45	188
5/4/2022	Episode 12: Deborah Seltzer Talks Coding and Billing for Substance Use Disorders (feat. Deborah Seltzer)	51	122
6/13/2022	Episode 13: SUD Treatment for Justice-Involved Patients | Day One Program (feat. Radha Sadacharan, MD, MPH and Rebecca Lee)	60	149

a Not available.

### CE Credit

**Figure 1.**
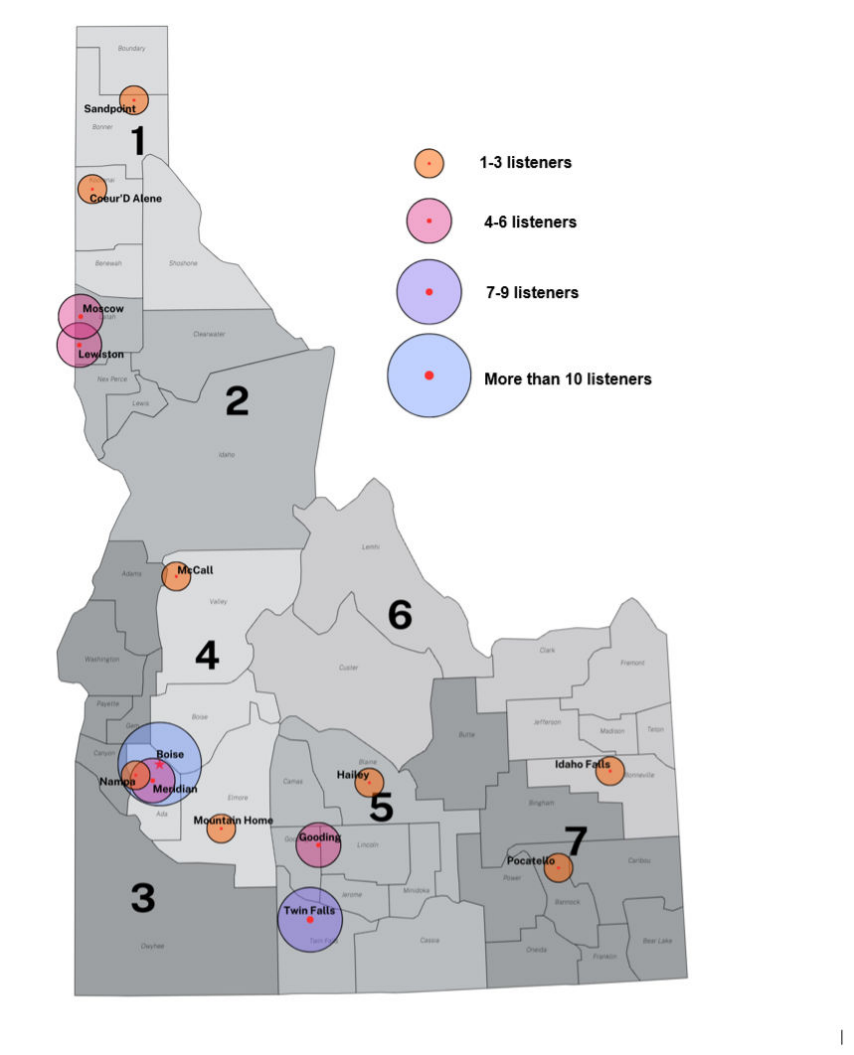
Health care professionals claiming continuing education credits from the ECHO Idaho “Something for the Pain” podcast within the state of Idaho. Values 1-7 represent Idaho’s 7 public health districts (map adapted from MapChart).

A total of 63 unique health care professionals representing 14 distinct cities and spanning all 7 public health districts within the state of Idaho, as well as out-of-state listeners, claimed CE credit for season 1 of the podcast (Figure 1). The podcast drew a diverse array of professionals from various disciplines, with social workers being the most widely represented group among those who claimed CE credits (Multimedia Appendix 1). Three questions were generated for each of the 13 episodes eligible for CE credits. The average percentage of correct answers for all episodes was 83% (SD 11.6%) (Multimedia Appendix 2), thus providing preliminary evidence related to audience engagement and potential impact on learning outcomes.

## Discussion

### Principal Findings

ECHO Idaho’s “Something for the Pain” podcast included 14 episodes providing best practices and resources for behavioral health, opioid use disorder, and SUD prevention, treatment, and recovery specific to Idaho. The podcast included interviews with health care professionals and community members who had first-hand experience and knowledge of evidence-based practices, as well as didactic presentations taken from previously recorded ECHO Idaho sessions. Audience recruitment primarily focused on health care providers and community members interested in the behavioral health and SUD field. Metrics such as click metrics, CE credits, and user experience questions were used to track listener engagement. Data collected from Eeds and iECHO systems were analyzed using SPSS, providing a descriptive statistical analysis.

The ECHO Idaho podcast effectively engaged a diverse group of health care professionals throughout Idaho, demonstrating the utility of podcasts as a versatile tool for professional development and outreach. This highlights the possibility of leveraging podcasts to not only provide CE but also to serve as a means of attracting more health care professionals to Project ECHO sessions. The widespread accessibility of podcasts suggests their potential as a potent recruitment tool for future Project ECHO initiatives.

This report presents a preliminary assessment of an innovative platform to engage with health care professionals, as well as community members, by providing insightful information and professional development material via the medium of podcasts.

### Limitations

Although the initial season of the podcast engaged with diverse health care providers across the state, the podcast’s impact on clinical practice remains unknown. Previous literature has suggested internet-based continuing medical education can increase knowledge, change physician behavior, and indirectly impact clinical practice [16,24]. Therefore, future research should collect feedback from nonlisteners and previous listeners to understand their preferences for this innovative platform as well as assess the direct impact of podcast-based CE on listeners’ knowledge and professional and clinical practice through quantitative and qualitative analyses.

Additionally, although several data analytics methods were used, some challenges remain. The RSS data records clicks (ie, listens) on each episode, but there is no way to track how long someone listened or if they completed the full episode. Therefore, interpretation of those data points should be made with caution. Similarly, due to data constraints, it is difficult to assess the benefits of the ECHO podcast compared to the more traditional ECHO program model. As a result, we were unable to fully evaluate the podcast’s impact, particularly in terms of reach, impact on learning outcomes, and its influence on clinical practice. Future research should focus on assessing these variables, as they are crucial for understanding the effectiveness of this type of CE material. Lastly, the effectiveness of our recruitment strategies remains undetermined. Future research should prioritize evaluating these strategies to optimize the return on investment and enhance the overall impact of the podcast.

### Lessons Learned

Overall, the goal of the podcast was to provide clinicians across the state with an easily accessible CE program. We learned that podcasts could be used to engage Idaho health care providers and provide access to CE opportunities. Although we provide evidence of successfully reaching listeners, a robust marketing campaign was not used. Furthermore, the topics were selected by a team of subject matter experts without input from listeners or previous ECHO Idaho attendees. In the future, assessing additional indicators (eg, listener preferences related to episode length, topic selection, impact on clinical practice, perceived barriers to listening to podcasts) could ensure that new podcast series and episodes meet the needs of current and potential listeners.

### Conclusions

The ECHO Idaho “Something for the Pain” podcast was developed as a new and innovative way of providing CE for health care providers. We provide evidence that this approach was successful in its efforts to engage a sizable audience of listeners who claimed CE credits for participation. The podcast had listeners from diverse health care professions representing cities from across Idaho and the United States. Future research efforts should include the collection of additional information such as listener preferences, knowledge change, professional and clinical impact, and patient outcomes to guide the implementation of Project ECHO information into an effective CE podcast format.

## Supplementary material

10.2196/55313 Multimedia Appendix 1 Unique health care professionals claiming continuing education credit from the ECHO Idaho “Something for the Pain” podcast.

10.2196/55313 Multimedia Appendix 2 Percentage of correct answers for questions of continuing education credit–eligible episodes.

## Abbreviations

CE: continuing education
ECHO: Extension for Community Health Outcomes
SUD: substance use disorder
WWAMI: Washington, Wyoming, Alaska, Montana, Idaho
