# Supplementary material for: Creation of the ECHO Idaho Podcast: Tutorial and Pilot Assessment
Source: JMIR Med Educ. 2025 Mar 21;11:e55313. doi: 10.2196/55313 (PMC11951813; doi:10.2196/55313)
Supplement: Multimedia Appendix 1 [file mededu-v11-e55313-s001.docx]

Multimedia Appendix 1

Unique healthcare professionals claiming CE credit from the ECHO Idaho “Something for The Pain” Podcast.

| **Profession (credentials)** | **Number** | **Percentage** |
| --- | --- | --- |
| Social Worker (LMSW, LCSW, LSW, CCM, BSW, MSW, CSW) | 22 | 35% |
| Nurse (RN, BSN, MSN, PhD, CNM, CNS, CRNA) | 7 | 11% |
| Counselor (LMFT, LCPC, LPC, CADC, ACADC, LP, PhD) | 6 | 10% |
| Nurse Practitioner (NP, DNP, APRN) | 5 | 8% |
| Physician (MD, DO) | 3 | 5% |
| Physician Assistant (PA, PA-C) | 3 | 5% |
| Psychologist (PhD, PsyD) | 1 | 2% |
| Public Health Professional (PhD, CHWs, CHES, DHSc, Dph, JD, EdD) | 1 | 2% |
| Other (e.g., healthcare related student, RD, CPA, EMT, CPC, etc.) | 15 | 25% |
