# Supplementary material for: Creation of the ECHO Idaho Podcast: Tutorial and Pilot Assessment
Source: JMIR Med Educ. 2025 Mar 21;11:e55313. doi: 10.2196/55313 (PMC11951813; doi:10.2196/55313)
Supplement: Multimedia Appendix 2 [file mededu-v11-e55313-s002.docx]

Multimedia Appendix 2

Percentage of correct answers for questions of CE credit eligible episodes.

| **Episode** | **Percent Correct** |
| --- | --- |
| Episode 1 | 95% |
| Episode 2 | 67% |
| Episode 3 | 90% |
| Episode 4 | 79% |
| Episode 5 | 65% |
| Episode 6 | 77% |
| Episode 7 | 95% |
| Episode 8 | 90% |
| Episode 9 | 97% |
| Episode 10 | 79% |
| Episode 11 | 80% |
| Episode 12 | 67% |
| Episode 13 | 93% |
